# Novel therapeutic agents for cutaneous T-Cell lymphoma

**DOI:** 10.1186/1756-8722-5-24

**Published:** 2012-05-17

**Authors:** Salvia Jain, Jasmine Zain, Owen O’Connor

**Affiliations:** 1 NYU Cancer Institute, Division of Hematology and Medical Oncology, NYU Langone Medical Center, New York, NY, 10016, USA; 2 Center for Lymphoid Malignancies, The New York Presbyterian Hospital - Columbia University Medical Center, Columbia University Hospital - College of Physicians and Surgeons, 6 East 60th St., New York, N.Y, 10022, USA

## Abstract

Mycosis fungoides (MF) and Sezary Syndrome (SS) represent the most common subtypes of primary Cutaneous T-cell lymphoma (CTCL). Patients with advanced MF and SS have a poor prognosis leading to an interest in the development of new therapies with targeted mechanisms of action and acceptable safety profiles. In this review we focus on such novel strategies that have changed the treatment paradigm of this rare malignancy.

## Introduction

Cutaneous T-cell lymphomas (CTCL) are a rare heterogeneous group of non-Hodgkin lymphomas derived from skin-homing mature T-cells. Mycosis fungoides (MF) and Sezary Syndrome (SS) represent the most common subtypes of primary CTCL, with an incidence rate of 4.1/1,000,000 person-years and male predominance [1]. The prognosis of MF and SS depends on the age at presentation, type and extent of skin lesions, overall stage and presence or absence of peripheral blood involvement and extra-cutaneous disease. In 2007, a revised staging system of MF and SS by the International Society for Cutaneous Lymphomas (ISCL)/European Organization for Research and Treatment of Cancer (EORTC) was proposed, incorporating stratification of early stage (TI/T2) into patch alone and both patch and plaque disease, as well as detailed histologic and molecular classification of lymph node and peripheral blood involvement leading to a uniform and standardized staging and classification system [2]. Agar et al. have validated this new staging system by analyzing the outcome of 1502 MF and SS patients treated at their institution [3]. In this study the median reported follow–up period was 5.9 years (range, 0.4–35.5 years). Seventy one percent of the patients had ‘limited-stage’ disease (stages IA, IB and IIA) with median overall survival (OS) ranging between 15.8 to 35.5 years whereas 20 percent of the patients had ‘advanced–stage’ disease (stages IIB, III and IV) and their OS was inferior ranging between 1.4-4.7 years. This retrospective analysis confirmed the previously observed dismal median OS of patients with SS (7% patients in this study were diagnosed to have SS), which was noted to be 3.1 years in this study from the time of diagnosis [4]. Owing to the heterogeneity and rarity of this neoplasm, there are few randomized trials to support treatment recommendations and step-wise treatment algorithms in various stages of CTCL, particularly advanced stage. Hence choice of treatment is often determined by physician or patient preference and is based on several factors, including stage of disease, use of previous therapies, availability and side-effect profile of the treatment, duration of response, patient convenience and expense. Treatment decisions vary considerably across both US and Europe. Hence the National Cancer Center Network (NCCN) and EORTC have published guidelines for the management of this malignancy that are based on consensus statements rather than evidence-based data [5,6]. Most experts tend to start with a single modality or agent of treatment and add additional therapies at the time of progression based on consensus recommendations or institutional and individual experience. Patients with limited stage disease are effectively treated with skin-directed therapies; these include topical nitrogen mustard, corticosteroids, bexarotene, localized radiotherapy or psoralen plus ultraviolet A therapy, as listed in the NCCN and EORTC guidelines. Most patients will achieve short-term clinical response but will have recurrent disease for many years but still have a normal life expectancy [5-7]. Recurrent disease after a durable remission can often be retreated with the same modality. If skin-directed therapies are ineffective or if the patient develops advanced stage disease then systemic therapies are introduced, which may include α-interferon, bexarotene, photopheresis, denileukin diftitox, vorinostat, alemtuzumab, cytotoxic chemotherapy, or combination therapies. In advanced stage disease, responses are often partial and seldom durable and there is no treatment for which improved survival has been demonstrated [3,4,8]. Allogeneic stem cell transplant is reserved for selected young patients with advanced-stage disease in order to exploit a graft versus lymphoma effect for long-term disease control. Since most patients require prolonged therapy, the key to successful disease management is to enhance immune function and minimize immunosuppressive therapies in order to reduce life threatening infectious complications. This has led to development of new therapies with targeted mechanisms of action and acceptable safety profile that may change the paradigm of treating this disease. Furthermore, clinical trials in MF and SS have suffered from lack of standardized criteria to assess response and clinical end points. This has made interpretation of clinical trials involving various agents performed to date cumbersome. The following consensus guideline was recently proposed to assess a uniform composite response based on the ISCL, USCLC and EORTC recommendations to facilitate collaboration among investigators: complete remission was defined as (100% clearance of all skin lesions + all lymph nodes ≤ 1.5 cm in long axis + normal size of visceral organs by imaging + ≤ 5% sezary cells in peripheral blood) while partial remission was defined as [50–99% clearance of skin lesions, + ≥ 50% reduction in SPD (sum of the product of the longest bidimensional diameters) + ≥ 50% regression of measurable disease in the organs + > 50% decrease in blood tumor burden] [9].

### Novel therapeutic agents in cutaneous T-cell lymphoma

While the lack of insight into the biology of CTCL has hindered the development of true ‘targeted therapies’, there is now an abundance of new drugs that have shown potentially significant activity either alone or in combination with conventional agents.

### Histone deacetylase inhibitors (HDAC inhibitors)

Histone deacetylase inhibitors have become a critical component of the CTCL treatment armamentarium in the relapsed/refractory setting. These are epigenetic agents that regulate gene transcription by physical alterations of either DNA or the structural components of chromatin. There are 18 HDACs (histone deacetylases) discovered in human cells that are divided into four classes based on their homology to HDACs found in yeasts. Class I, II and IV HDACs require zinc for their enzymatic activity, whereas class III HDACs, also known as silent informant regulator proteins (Sirtuins), require NAD^+^ as a coenzyme for their activity. HDAC inhibitors are a chemically broad group of molecules found to inhibit the activity of HDACs in a wide range of concentrations from low nM to high mM. To date, there are several HDAC inhibitors in development with focus on greater potency and improved tolerability. The bulk of the clinical experience with these compounds has focused on the pan-HDAC inhibitors (i.e., those inhibiting class I, II and IV enzymes). By causing accumulation of acetylated histones and non-histone proteins in the cell, HDAC inhibitors lead to several effects, including transcriptional modification and altered function of proteins regulating cell proliferation, cell cycle progression, differentiation and apoptosis [10,11]. HDAC inhibitors can block cell proliferation and cause apoptosis in human tumor derived cell lines by causing cell cycle arrest in G1 or G2/M phase with relative sparing of normal cells through dysregulation of proteins that control cell cycle progression and coordinate the G1/S and G2/M transition such as cyclins, cyclin-dependent kinases (Cdk) and their associated regulators [12-14]. HDAC inhibitors can also induce upregulation of p21, p27 and p16, which bind to and inactivate CDK2 and CDK4, hence leading to inhibition of cell cycle progression [15,16]. HDAC inhibitors have also been observed to increase the expression of genes that encode for death receptors and their ligands, such as Fas and the Apo 2 L/TRAIL receptors, DR4 and DR5, downregulate c-FLIP, c1AP2 and X1AP and induce generation of reactive oxygen species (ROS) all of which contribute towards apoptosis [17-22]. Other non-histone targets of HDAC inhibitors include transcriptional co-regulators, DNA binding transcriptional factors, chaperone proteins, steroid receptors and DNA repair enzymes [23-25]. HDAC inhibitors have been shown to have antiangiogenic effects by various mechanisms, including up-regulation of angiogenesis inhibitors such as thrombospondin and von-Hippel Lindau factor, as well as downregulation of factors that promote vasculogenesis, such as VEGF and hypoxia-induced protein (HIF-[alpha]) [26,27]. Marquard et al. investigated HDAC expression and histone acetylation by immunohistochemical staining in 73 patients with CTCL [28]. The expression of HDAC1, HDAC2, HDAC6, and acetylated H4 were correlated to histological subtypes and overall survival. HDAC2 and acetylated H4 were more common in aggressive forms of CTCLs compared with indolent CTCLs whereas HDAC6 was associated with a favorable outcome. Despite these many pleiotropic effects, it has been difficult to assign a precise mechanistic basis to any one or more of these drugs in any particular tumor type. As single agents these drugs appears to have a class effect in T-cell lymphomas and CTCL in particular. A brief description of the various HDAC inhibitors currently in clinical use or trials for CTCL treatment is listed below. Two agents of this class, vorinostat (Zolinza) and romidepsin (FK 228, Depsipeptide, Istodax) are approved for the treatment of relapsed or refractory CTCL in the US [29,30].

#### Vorinostat (Zolinza)

Vorinostat (suberoylanilide hydroxamic acid), is an oral pan HDAC inhibitor (inhibits class I, II and IV of HDACs) that was approved by the U.S. FDA for the treatment of patients with relapsed or refractory CTCL in October 2006 based on response rates [29]. The mean terminal half- life is 1.45 h. In vitro studies in CTCL cells demonstrated anti-proliferative action with the half-maximal inhibitory concentration IC_50_ to be in micromolar range with selective induction of apoptosis of the transformed T-cells [31]. Kelly et al. conducted a phase I trial of intravenously administered vorinostat in a variety of hematological and solid malignancies and based on grade 3 and 4 leukopenia and thrombocytopenia, 300 mg/m2/day for 5 days for 3 consecutive weeks was established as the maximum tolerated dose (MTD) in patients with hematologic malignancies [32]. O’Connor et al. conducted two consecutive phase I trials to compare the activity and toxicity of oral and intravenous formulations of vorinostat [33]. Regardless of the formulation, vorinostat was well tolerated in patients with relapsed/refractory lymphomas although the toxicity was profile was different with fatigue, anorexia, diarrhea and dehydration being the dose limited toxicities (DLTs) for patients on oral vorinostat and myelosuppression being the DLT for patients receiving intravenous vorinostat. This led to the first single center phase II trial of vorinostat in CTCL by Duvic et al. where 33 advanced, heavily pre-treated CTCL patients received 3 different dosages and schedules of vorinostat including 400 mg daily, 300 mg twice daily for 3 days followed by 4 days of rest every week for 4 weeks followed by 5 days every week and 300 mg twice daily for 14 days with a week of rest followed by a maintenance dose of 200 mg twice daily [34]. Treatment was continued until the patient showed signs of progression or toxicity. Based on response and toxicity profile a dose of 400 mg daily was identified as the optimal dose for this patient population. The overall response rate (RR) was 24.4% with 8 of the 33 patients having partial remission (PR) including 4 of 11 patients with SS. More importantly, 14 of the 33 patients (42%) reported significant relief from pruritus. The median time to response was 11.9 weeks, while the median overall response duration was 15.1 weeks. This dose was chosen for the subsequent pivotal multicenter phase IIB trial of 74 patients conducted by Olsen et al. in relapsed CTCL which showed an overall response rate of 29.7%, [(22 of 74, of which 21 achieved PR and 1 achieved complete remission (CR)] with and a 29.5% (18 of 61) response rate in patients with ≥ Stage IIB disease [35]. Ten of 30 patients with SS (33%) demonstrated clinical responses. The median time to objective response was 56 days, though some patients took up to 6 months to respond. A Post–Hoc analysis of 18 patients in the trial with high blood tumor burden revealed objective blood response in 28% patients (5/18) and an objective skin response in 44% (8/18) patients [36]. The most common drug-related side-effects observed during clinical trials included fatigue, GI symptoms (nausea, vomiting, diarrhea), hematologic (especially thrombocytopenia and anemia), dysgeusia, anorexia, weight loss and spasms. Thromboembolism was reported in 5% of patients and corrected QT interval prolongation was reported albeit of no clinical relevance. The ease of schedule, route of administration and the low toxicity profile of vorinostat led to evaluation of combination therapies. Gardner et al. in their case series of 3 patients reported a clinical response in 1 SS patient when vorinostat was combined with interferon gamma and photopheresis [37]. Patient had a durable response of 14 months. Vorinostat is a very promising agent in the treatment of CTCL and is currently in multiple clinical trials exploring its combination with other agents such as bortezomib, lenalidomide and combination chemotherapy.

#### Romidepsin

Romidepsin a cyclic peptide originally isolated from the broth culture of *Chromobacterium violaceum* and was approved for the treatment of CTCL patients who have failed at least 1 prior systemic therapy in November 2009. It is a potent pan HDAC inhibitor (inhibits class I, II and IV of HDACs) with a terminal half-life of 2.64 h and is administered intravenously. In vitro studies have demonstrated cytotoxic effects of romidepsin in CTCL cell lines with the half–maximal inhibitory concentration IC_50_ to be in the nanomolar range in comparison with micromolar range IC_50_ of vorinostat and belinostat [38]. The first phase I clinical trial of romidepsin was conducted in 2001 at the NCI by Piekarz et al. and included four patients with refractory T-cell lymphomas (2 patients with SS, 1 patient with tumor-stage CTCL, and 1 patient with unspecified peripheral T-cell lymphoma) [39]. Both patients with SS who were refractory to conventional chemotherapy agents had a rapid decrease in the percentage of circulating Sézary cells along with improvement in the skin erythema and edema. There was rapid clearing of all tumors after six cycles of treatment with romidepsin in the third patient. This promising activity led to conduction of two major independent multicenter phase II trials of romidepsin in patients with pretreated CTCL; the pivotal, Gloucester Pharmaceuticals initiated GPI-04-0001 and the National Cancer Institute (NCI) sponsored, NCI-1312 study [40,41]. The two trials differed in their enrollment criteria and efficacy assessment tools but romidepsin was administered as a 4-hour intravenous infusion at the dose of 14 mg/m2 on days 1, 8, and 15 of a 28-day schedule in both studies. In the NCI-1312, phase II investigator initiated study, 71 MF/SS patients who had no more than 2 prior cytotoxic chemotherapy regimens were enrolled with majority patients having ≥ stage IIB (87.3%) and 12.7% patients having stage IA-IIA. The overall RR for the group of 71 patients was 34% (24/71) with 4 (7%) CRs and 20 (26%) PRs. Stable disease was noted in 26 patients (38%). The median duration of response (DOR) was notable at 13.7 months (range, 1–66+ months). Among the patients with a major response (complete or partial), the median time to response (TTR) was 2 months (range, 1–6 months). The median time to progression (TTP) was 15.1 months for patients with a major response and 5.9 months for patients with stable disease. One of major criticisms of this trial was the choice of response assessment tools. Disease in skin or viscera was assessed by Response Evaluation Criteria in Solid Tumors (RECIST) criteria, lymph node involvement was assessed using International Working Group Guidelines (IWGG), and bone marrow involvement was recorded as present or absent, which were considered to give inaccurate estimates of disease burden in skin or blood [42,43]. This was corrected in the pivotal, registration study, GPI-04-0001 which adopted the more comprehensive SWAT score for response assessment or in the case of erythroderma, the 5-point erythroderma score [44-47]. Of the 96 patients enrolled in the GPI-04-0001 study, 29.2% had stage IB-IIA and 70.8% had ≥ stage IIB similar to the NCI study. Despite difference in tools adopted by the two studies for response assessment, results of the GPI-04-0001 study were remarkably similar to the NCI study which included an overall RR of 34% (33/96) including 6% CR (6/96), median TTR of 1.9 months, median time to progression (TTP) of 8.3 months and median DOR of 14.9 months. One of the striking features of both studies was the long duration of response achieved in most patients, extending beyond 3 years, in some. Based on the results of these two single arm studies, romidepsin was given final approval by the FDA for use in relapsed/refractory CTCL. Overall romidepsin was well tolerated in both studies. Nausea (all grades, 52%–56%), asthenia (all grades, 44%), anorexia (all grades, 20%), vomiting (all grades 19%–26%), and aguesia (all grades, 13%) were commonly seen in patients receiving romidepsin. Most of these adverse events were mild (grade 1 or 2) and were very manageable. Constitutional symptoms of fatigue (all grades, 41%) and fever (25%) have been observed in patients on romidepsin treatment and has been hypothesized by some to be cytokine mediated based on the increased levels of IL-6 detected in post treatment samples of at least two patients treated with belinostat who had reported severe fatigue [48]. Lymphopenia and granulocytopenia, regardless of causality, occurred in 3% and 8% of patients in the GPI-04-0001 study versus 55% and 52% of patients in the NCI study. Infection was reported in 45% of cases in both studies; however, most of these were attributable to disease and not related to romidepsin. Although asymptomatic T-wave flattening was common in the NCI study, the results of an intensive safety analysis revealed no clinically significant corrected QT interval prolongation or electrocardiographic abnormalities attributable to romidepsin [49]. Despite these findings from the intensive analysis of cardiac toxicity data, the package insert dictates that the drug needs to be administered with caution in patients with significant preexisting cardiac abnormalities and concomitant medications that prolong QT interval or inhibit CYP3A4 are generally avoided. A topical formulation of romidespin is currently in clinical trials in limited stage CTCL.

#### Belinostat

Belinostat (PXD101) is a hydroxamate pan-histone deacetylase inhibitor (inhibits class I, II and IV HDACs) which demonstrates broad anti-neoplastic activity in vitro with half –maximal inhibitory concentration IC_50_ in CTCL cell lines in micromolar range. Its half-life ranges between 0.3-1.3 h for intravenous formulation and 1.8-1.9 hours for the oral formulation. Two parallel phase I studies were conducted to determine the MTD of the intravenous formulation in refractory solid tumors and hematologic malignancies [48,50]. In the 46 patients with advanced solid cancers that received intravenous belinostat, MTD was established at 1000 mg/m2/d for 5 days every 3 weeks based on DLTs of grade 3 fatigue, diarrhea and atrial fibrillation [48]. However, pharmacodynamic effects of belinostat on histone acetylation were most notable in the first few hours following intravenous drug administration. Consequently, it was hypothesized that a protracted or continuous daily oral dosing schedule could be advantageous, allowing continual target inhibition leading to an evaluation of oral belinostat in an extension of this study mentioned later in this section. Stabilization of disease was observed in a total of 5 of 16 patients (2 patients with diffuse large-cell lymphoma, two patients with chronic lymphocytic leukemia and 1 myeloma patient) enrolled in the phase I study by Gimsing et al.which led to its investigation in T-cell lymphomas [50]. An extension of the previously reported phase I study by Steele et.al. [48] was performed to explore the feasibility of oral belinostat [51]. In this study, 15 patients who had previously received intravenous belinostat were administered oral belinostat in a range of doses (900–1000 mg/m2) and schedules (once, twice or thrice daily), on either day 1 or days 1–5 every 3 weeks. Doses as high as 1000 mg/m2 for 5 consecutive days was tolerated. The estimated fasting bioavailability was 15 – 35%. Of interest, level of target inhibition (i.e. histone acetylation) was similar to that achieved by intravenous route. Oral dosing was fully assessed in a further study of 92 patients where a variety of schedules were investigated [52]. Based on DLTs, the recommended phase II doses for oral formulation were: (i) 250 mg daily and twice a day for 28 days (ii) 750 mg on days 1 – 14, every 21 days; and (iii) 2000 mg on days 1 – 5, every 21 days. Further dose escalation studies beyond 2000 mg are ongoing and the highest dose for the day 1–5 dosing is yet to be determined. A multicenter phase II trial by Pohlman et al. evaluated the activity of belinostat in patients with PTCL or CTCL who had failed 1 prior systemic therapy. Belinostat (1000 mg/m [2]) was administered as a 30-min IV infusion on days 1–5 of a 3 week cycle [53]. Twenty nine patients in the CTCL arm (including 15 MF, 7 SS, 5 non-MF/SS and 2 unclassified) were enrolled with majority (55%) having stage IV disease and were treated for a median of 2 cycles (range, 1–6). Four responses were observed (14%) with 2 CR (Anaplastic large cell lymphoma and MF), 2 PR (MF and SS) and 16 stable-disease (SD). The median duration of the response was 273 days (range, 48–469+). Pruritus relief was noted in 7 out of 14 patients. Of interest median time to response and relief of pruritus was short of 16 days. Overall in both study arms the drug was well tolerated and most adverse events were only grade 1/2 (nausea, fatigue, constipation, diarrhea, and vomiting). There were 4 grade 3/4 AEs that included peripheral edema, adynamic ileus, pruritus and rash. No significant hematologic or cardiac toxicity was observed. Belinostat’s activity as monotherapy has been most marked in the lymphoid malignancies and in particular, relapsed or refractory PTCL and CTCL; as a result the FDA granted belinostat orphan drug status for PTCL, and a pivotal phase II registration trial (the BELIEF study) for this tumor type is underway.

#### Panobinostat

Panobinostat, also known as LBH589, is an oral pan-HDAC inhibitor (inhibits class I, II and IV HDACs) belonging to the hydroxamic acid group that has shown activity in patients with CTCL in a phase I and II trials [54,55]. It has a long half-life of 8 h and exerts potent cytotoxic effects in CTCL cell lines with an IC_50_ in nanomolar range similar to romidepsin. Duvic et al. conducted an open label multicenter phase II trial with the primary objective of establishing the efficacy and safety of panobinostat for patients with relapsed/refractory CTCL with Stage IB–IVA MF and SS [55]. Ninety five patients (70 with MF and 25 with SS) were enrolled who had received 3–4 median prior treatment regimens and mostly were ≥ Stage IIB at study entry. They received a median of 3 (range, 1–17+) treatment cycles of panobinostat. Eleven out of 62 patients who had received prior bexarotene therapy had confirmed skin responses by modified SWAT (mSWAT), including 2 complete skin responses and were awaiting CT scans to assess systemic response. Four of the 33 bexarotene naïve patients had confirmed skin and CT scan responses. Common adverse events (>20%; all grades regardless of causality) included diarrhea, thrombocytopenia, nausea, pruritus, fatigue and asthenia while Grade 3/4 AEs (>2%, regardless of causality) included thrombocytopenia, neutropenia, pruritus, diarrhea, and hypophosphatemia. Based on this trial, panobinostat demonstrated encouraging efficacy with good tolerability in MF and SS patients potentially making it an attractive alternative HDAC inhibitor in the treatment of CTCL. A mechanistic study of panobinostat showed that this agent induces CXCR4 degradation via the proteasomal pathway and has synergy with CXCR4 inhibition, supporting the rationale for testing such a combination in vivo [56]. It is being evaluated in several hematologic malignancies including T-cell lymphomas in combination with conventional chemotherapy agents, proteasome inhibitor, bortezomib and m-TOR inhibitors, everolimus and RAD001.

### Future direction of HDAC inhibitors in CTCL

The biological basis for the remarkable clinical activity of HDAC inhibitors in CTCL remains elusive. We anticipate more scientific and translational studies to dissect the molecular mechanisms behind this high efficacy and favorable tolerability. Given the plethora of HDAC inhibitors demonstrating promising but variable activity in CTCL, we anticipate that future clinical trials of HDAC inhibitors will incorporate detailed molecular analysis of the tumors to identify predictive biomarkers of response or resistance to individual agents or as a class to enable delivery of personalized therapy. One such example is the increased expression of phospho-NF-ĸB p65 and phospho-STAT-1 demonstrated in a vorinostat resistant cell line (called as HH/VOR, generated by exposing the vorinostat sensitive HH cell line to increasing concentations of vorinostat) [57]. Using a genome-wide loss of function screen with a short hairpin RNA (shRNA) library, HR23B, a component of the proteasome, was identified as a determinant of HDAC inhibitor sensitivity [58]. HR23B is expressed at high levels in CTCL, and there was a correlation between HR23B expression and clinical response to HDACis [59]. Presently there are no trials comparing one HDAC inhibitor with another and hence while romidepsin appears most promising it cannot be definitively concluded that it is superior to other HDAC inhibitors. Of note, unpublished anecdotal evidence suggests lack of cross-resistance across the spectrum of HDAC inhibitors in the treatment of CTCL. However this needs prospective validation in a randomized clinical trial. Currently, HDAC inhibitors can be used as single agents after the failure of at least one to two systemic therapies for as long as there is clinical benefit or as long as the patient can tolerate the treatment. Most physicians like to use them before use of chemotherapy agents given the high efficacy and acceptable tolerability. Unfortunately at the moment there are no clinical guidelines on how to use them best during the course of this chronic malignancy. As data regarding HDAC inhibitor combinations with conventional chemotherapy agents, hypomethalating agents, proteasome inhibitors and radiation become available; it is likely that the addition of another agent at progression will become a standard of care.

### Anti-folate antagonist

#### Pralatrexate (Fotolyn)

Pralatrexate (PDX, 10-propargyl 10-deazaaminopterin), a novel antifolate designed to have high affinity for the reduced folate carrier (RFC) was approved for the treatment of relapsed or refractory PTCL in September 2009 based on the pivotal multicenter international registration study PROPEL which demonstrated an overall RR of 29% (32/109), including 12 (11%) CRs and 20 (18%) PRs [60]. Pralatrexate is also a more effective substrate for polyglutamation by FPGS so it is more efficiently polyglutamated compared to methotrexate which subsequently leads to higher intracellular accumulation of the drug compared to methotrexate which also enhances its affinity for dihydrofolate reductase (DHFR) [61-63]. Analysis of 12 patients with refractory transformed MF in the PROPEL study demonstrated a 58% objective RR using International Workshop Criteria [64]. The significant activity observed in this group led to the dose finding multicenter study that initially enrolled 31 patients with relapsed/refractory MF, SS and primary cutaneous anaplastic large–cell lymphoma into 6 cohorts at varying dose and schedule and established the optimal dose and schedule as 15 mg/m2 weekly for 3 out of 4 weeks based on grade 3 mucositis [65]. Based on the mSWAT score, an overall RR of 43% (12/28) was observed at the above stated optimal dose. This highlighted promising activity of this agent and the toxicity profile suggested that it could be given for prolonged time periods to sustain responses. Toxicity was mild and consisted of grade 1 and 2 adverse events (AEs) included fatigue (34%), mucositis (28%), nausea and edema (24%), epistaxis (21%), pyrexia (17%), anemia (14%), etc. A phase II trial of pralatrexate in combination with bexarotene in relapsed/refractory CTCL is ongoing. Recently Marchi et al. demonstrated synergy of pralatrexate with proteasome inhibitor bortezomib in preclinical models of T-cell lymphoma laying the platform for development of further combination therapies of pralatrexate with other active agents T-cell lymphoma [66].

### Proteosome inhibitors

#### Bortezomib (Velcade)

The proteasome inhibitor bortezomib is a dipeptide boronic acid analog that reversibly inhibits the chymotryptic activity of the 20S subunit of the proteasome. Bortezomib targets the catalytic 20S core of the proteosome with a multitude of downstream effects including inhibition of cellular protesosome, accumulation of the cell cycle-dependent kinase inhibitors such as p27/p21, activation of p53, inhibition of NF-kB and accumulation of pro-apoptotic proteins like Noxa which inactivates anti-apoptotic proteins like McL-1 [67]. Bortezomib is the first agent of this class to be approved for the treatment of multiple myeloma and mantle cell lymphoma [68,69]. It has been hypothesized that cancer cells are more dependent on the proteasome for clearance of abnormal or mutant proteins [70]. In fact, several preclinical studies have shown that malignant cells are more sensitive to proteasome inhibition than normal cells [71-75]. Bortezomib has an elimination half-life of 9 to 15 h. Zinzani et al. conducted a phase II trial of bortezomib (1.3 mg/m2 on days 1,4, 8, and 11 of a 21-day cycle) in 15 patients with relapsed/refractory T-cell lymphoma, including PTCL (2 patients) or CTCL (10 patients all MF, no SS) with a reported overall response rate (ORR) of 67% (with 2 CR’s and 6 PR’s) [76]. Among the CTCL patients who received a median of 6 cycles, there was a RR of 70% (7 of 10) to bortezomib. One CR (10%) lasted for more than 12 months and 6 PR (60%) were observed. The responses were also durable in CTCL patients, ranging from 7 to 14 months even in patients with PR’s. The most common dose limiting side-effects with bortezomib are myelosuppression (neutropenia and thrombocytopenia) and sensory neuropathy that occurred in 30-40% patients with myeloma and 50% of patients with CTCL treated with bortezomib. Most patients had improvement or resolution of symptoms off drug. A phase I study evaluated the use of standard dose CHOP (cyclophosphamide, doxorubicin, vincristine, and prednisone) plus bortezomib in 13 patients with advanced, aggressive T-cell or NK/T-cell lymphoma [77]. No dose limiting toxicities were observed up to the maximal dose of bortezomib of 1.6 mg/m2. The CR rate was 62%. No data were provided for progression free or overall survival. Thus while experience with the use of bortezomib in patients with CTCL is limited, the responses observed are durable and toxicity was acceptable. We anticipate that carfilzomib, the new selective irreversible epoxyketone 20S proteasome inhibitor which causes minimal neuropathy and demonstrates promising activity in both bortezomib naïve and treated myeloma patients will be soon evaluated in T-cell lymphoma patients including CTCL [78-80]. Several preclinical studies and early phase clinical trials have explored the feasibility, efficacy and toxicity of bortezomib and HDACis in several malignancies such as multiple myeloma, acute myeloid leukemia, myelodysplastic syndrome, mantle cell lymphoma, CLL and a host of solid tumors and demonstrated response with minimal toxicity. However given pleiotropic effects of both classes of agents it has been difficult to elucidate a precise biologic rationale for synergy. Nevertheless, there are multiple ongoing trials exploring the efficacy and toxicity of bortezomib in combination with multiple HDACis in CTCL and we hope that molecular co-relates from these trials will provide a mechanistic basis for the synergistic activity.

### Nucleoside analogs

#### Forodesine

Forodesine is a transition state purine nucleoside phophoryalse inhibitor (PNP). Inhibiton of this enzyme leads to T-cell selective intracellular accumulation of dGTP resulting in apoptosis and cell cycle arrest. An open-label phase I dose-escalation study of oral forodesine (40 to 320 mg/m [2]) given daily for 4 weeks was conducted by Duvic et al. [81]. The primary objective was to determine the maximum and optimal tolerated dose with assessment of response by mSWAT. Nine of an intended 64 patients were available for evaluation when the initial results were reported at the ASCO 2009 annual meeting. All 9 had completed > 12 months of treatment with ongoing responses in at least 3 of them ranging from 416–863 days. Of the 9 patients, 2 patients attained CR, 3 achieved a PR and 4 had stable disease (SD). The most common related adverse events were nausea (44%), fatigue, peripheral edema, dyspnea, and urinary casts (22%). Two of the 9 patients experienced treatment related grade 3 or higher peripheral edema and diffuse large B-cell lymphoma. Hematologic toxicity and infections related to the forodesine were not seen. Forodesine definitely shows acceptable tolerability in patients with T-cell lymphoma, is convenient in administration (oral) and has efficacy on extended duration of therapy (>12 months). A phase II study of forodesine monotherapy is ongoing in patients with relapsed and refractory CTCL patients.

### Monoclonal antibodies

#### Anti-CD52 (Alemtuzumab)

Alemtuzumab or Campath-1H is a recombinant DNA- derived humanized IgG_1_ kappa monoclonal antibody that is directed against CD52, a 21–28 kDa cell surface glycoprotein that is expressed on mature lymphocytes. Ginaldi et al. investigated the intensity of expression of CD52 in a study of 45 cases [24 cases of B-cell chronic lymphocytic leukemia (CLL), 21 cases of T-cell prolymphocytic leukemia (T-PLL) and 12 normal controls] and showed that normal T lymphocytes expressed higher CD52 expression than B lymphocytes and that the antigen was also significantly higher in T-prolymphocytic leukemia (T-PLL) compared with CLL [82].Of interest, the level of CD52 expression was somewhat higher in patients who responded to alemtuzumab than in non-responders which might provide a rationale as to why patients with T-PLL had better response in comparison with patients with CLL. Alemtuzumab is administered as an infusion over 2 h in a dose escalation manner starting at a dose of 10 mg three times a week and increased up to 30 mg thrice per week for a total of 12 weeks depending upon tolerability. Responses are evaluated at the end of the 12 weeks. Several reports of promising activity have been reported in patients with MF and SS albeit with small number of patients [83-87]. A phase II study was conducted by Lundin et al. which included 22 patients with refractory, ‘advanced-stage’, and CD52 positive CTCL with an overall RR of 55% (32% CR, 23% PR) including clearing of sezary cells in 6 out of 7 patients and better responses in erythrodermic patients over those with plaques or skin tumors [85]. There is a great deal of variability between definitions of SS in some of the earlier studies making difficult to interpret results. However, in studies where SS is clearly defined per the revised ISCL criteria, an overall RR between 82-100% with CR of 21–100% has been demonstrated with a DOR ranging from <3 months to > 12 months. Most of these studies are either retrospective, single institution based case series or phase II studies with very small number of patients treated (for example, n = 6). Despite the potent anti-tumor activity, profound immunosuppression secondary to this agent remains of significant concern and infectious complications including bacterial sepsis and CMV reactivation have been observed in two-thirds of treated patients with alemtuzumab. Careful monitoring for CMV reactivation and appropriate prophylaxis for PCP/HSV/VZV is recommended for patients treated with alemtuzumab. Subcutaneous (SQ) administration of this agent has also been explored to reduce toxicity and has been shown to demonstrate better tolerability with similar peak drug concentrations achieved but at a higher cumulative dose, and a higher potential to develop anti-alemtuzumab antibodies compared with intravenous dosing [88]. Alinari et al. further assessed the tolerability of SQ alemtuzumab in 5 elderly SS patients with multiple co-morbidities who were administered SQ alemtuzumab with dose escalation from 3–30 mg over 5–7 days followed by 30 mg thrice a week for 5–9 weeks [89]. An overall response rate of 100% with 4 unconfirmed CR’s and 1 complete CR (clearance of skin lesions also) was observed along with rapid relief from pruritus and rapid clearance of tumor cells from blood. No grade 3 & 4 hematologic toxicity was observed. Two asymptomatic CMV reactivations and one asymptomatic low-level EBV reactivation were noted both of which cleared with 2 weeks of oral valganciclovir or valacyclovir. While the number of patients in this report is small, it demonstrates that SQ administration of alemtuzumab is well tolerated despite advanced age and poor performance status. In another attempt to reduce the incidence of treatment related opportunistic infections, a phase II study by Zinzani et al. assessed the impact of a reduced dose (10 mg three times per week for four weeks) schedule in 10 patients with pretreated cutaneous/peripheral T-cell lymphoma [90]. Of these, 6 had nodal PTCLU and 4 had MF. All MF patients who were also co-treated with betamethasone were in stage T3 or T4. In the MF subset, the best response was a PR that was seen in 3 of the 4 patients (75%) with no ≥ grade 3 hematologic toxicity. Bernengo et al. further explored the efficacy of low dose SQ alemtuzumab in 14 SS patients (11 relapsed and 3 untreated) [91]. Four received 3 mg alemtuzumab on day 1, 10 mg on day 3, then 15 mg on alternating days. A reduced dosage (3 mg on day 1, then 10 mg on alternating days) was administered to the remaining patients. Overall, 12 of the14 patients (85.7%) achieved a clinical response, with 3 complete responses (21.4%) at a median follow-up of 16 months with TTF of 12 months. No patient in the group treated with 10 mg developed hematologic toxicity or infections while 28% patients experienced infectious complications at dose ≥15 mg. Thus this study demonstrated that low dose intermittent SQ alemtuzumab (10 mg maximum per administration) induced favorable outcomes with durable responses and benefit of reduced incidence of infectious complications making this an attractive option.

#### Anti-CD4

CD4 belongs to the immunoglobulin superfamily and acts as a co-receptor of TCR (T-cell receptor). It is normally expressed in helper T-cells, regulatory T-cells, macrophages, monocytes and dendritic cells and highly expressed by T-cell malignancies including PTCL’s and CTCL’s. Different monoclonal antibodies against T-cell antigens have been developed and evaluated clinically with limited success in T-cell lymphomas. Knox et al. reported results with SK3, a chimeric non-depleting anti-CD4 monoclonal antibody, in 7 patients with MF where the antibody was administered IV two times per week for 3 consecutive weeks at either the 10, 20, 40, or 80 mg dose [92]. No patient in the group treated with 10 mg developed hematologic toxicity or infections. Two objective responses were observed at the highest dose group. The trial was limited by the development of antichimeric antibodies. M-T412, an anti-CD4 chimeric antibody directed against a different epitope of the CD4 molecule, demonstrated a higher affinity and was able to induce CD4 positive lymphocyte depletion through an Fc-mediated mechanism. This was studied in 8 MF patients where patients received a single IV administration of 50, 100 or 200 mg and showed an objective response in 5 patients with freedom from progression lasting 25 weeks [93]. The antibody was well tolerated with clinical efficacy and low level of immunogenicity but with a weak signal of activity. One of the more promising anti CD4 antibodies is Zanolimumab (HuMax), a fully humanized anti-CD4 monoclonal antibody that is specific for the CD4 receptor expressed on most T lymphocytes and to a lesser extent on macrophages. Kim et al. conducted two identical prospective, multicenter, phase 2 studies (Hx-CD4-007 and Hx-CD4-008) in patients with relapsed/refractory CTCL in ‘early-stage’ and ‘advanced-stage’ respectively [94]. Thirty-eight patients with MF and 9 patients with SS were treated with 17 weekly infusions of zanolimumab (early-stage patients, 280 and 580 mg; advanced-stage, 280 and 980 mg) with assessment of response by CA score (composite assessment of index lesion disease severity). Thirteen objective responses were observed in the MF and 2 in the SS group respectively. Duration response was 12–24 weeks in the MF patients in the low dose group. High response rates up to 56% were noted in the high dose group with median response duration of 81 weeks. Most common treatment related AEs included inflammatory skin reactions and infections of the skin and upper respiratory tract. Of the 9 infections related to the drug, the majority was grade 2 or less and felt to be secondary to profound depletion of CD4^+^ lymphocytes. Thus zanolimumab demonstrated encouraging responses even though the duration of response is short. It is currently being tested in a pivotal phase III multicenter study in the US in MF and SS patients who are intolerant or refractory to bexarotene and one other standard therapy.

#### Anti-CD30

CD30 or the Ki-1 antigen is a cell surface leukocyte activation transmembrane protein of 120 kDa belonging to the TNF receptor superfamily that can be shed in a soluble form. It is expressed on mitogen activated B and T lymphocytes but not on resting monocytes and lymphocytes making it an attractive target. It is also expressed on malignant hematopoietic cells including Hodgkins lymphoma, ALCL, primary cutaneous ALCL, lymphomatoid papulosis and certain cases of transformed MF and is an attractive agent for therapy. Although the function of CD30 has not been clearly defined, it has been implicated in both cell death and proliferation [95]. In the normal physiologic state, CD30 cell surface expression is limited to activated T, B, and natural killer lymphocytes. Because CD30 serves as a diagnostic marker for the CD30 lymphoproliferative disorders, has a limited expression profile on normal cells and tissues, and has apoptosis-inducing characteristics, it has been evaluated as a target for immunotherapy [95,96]. Results of early generation naked monoclonal antibodies targeting CD30 have been disappointing due to their poor antigen binding properties, ineffective activation of effector cells, and neutralization by soluble CD30 [96-98]. SGN-30, is a humanized chimeric more potent CD30 monoclonal antibody with specificity for CD30 that is distinct from other anti-CD30 antibodies [95]. In preclinical studies, SGN-30 induced growth inhibition of Hodgkin’s lymphoma and ALCL cell lines in vitro and has shown potent antitumor activity in xenograft models of disseminated and localized Hodgkin’s lymphoma and ALCL [95]. It was evaluated by Duvic et al. in a multicenter phase II trial in heavily pretreated CD30-expressing cutaneous tumors including primary cutaneous anaplastic large cell lymphoma (pc-ALCL), lymphomatoid papulosis and transformed MF (t-MF) [99]. The initial dose was 4 mg/kg given every 3 weeks, which was later increased to 12 mg/kg. Of the 3 patients with t-MF, 1 achieved a PR, 1 demonstrated SD while one failed to respond. Duration of response in the patient achieving PR was 84 days and side-effects were minimal highlighting its antitumor activity and tolerability in CTCL patients.

#### SGN-35 (Brentuximab Vedotin)

SGN-35 is a drug-antibody conjugate in which SGN-30 is linked to monomethylauristatin E (MMAE), a synthetic antitubulin agent to enhance antitumor activity. After binding CD30, the conjugate is rapidly internalized inside the cell through CD30 receptor mediated endocytosis where it binds to tubulin and initiates cell cycle arrest. Two phase I studies have been conducted evaluating efficacy and tolerability of two different schedules (every 3 weeks or weekly). In the first dose-escalation trial conducted by Younes et al., 45 patients with refractory or recurrent CD30-positive hematologic malignancies, including HL (n = 42), systemic anaplastic large cell lymphoma (sALCL; n = 2), and angioimmunoblastic T cell lymphoma (n = 1) were treated on an every 3 week basis [100]. Approximately 75% of patients reporting B- symptoms at baseline experienced symptom resolution on study. Most patients (86%) had reductions in target lesion size. Among 28 evaluable patients treated at doses 1.2 mg/kg every 3 week, the objective response rate (CR + PR) was 46% (n = 13) and the complete remission rate was 25% (n = 7). Two additional PRs were observed in the 0.6 mg/kg cohort. Median response duration to date was 22 weeks (range, 0.1+ to 38+ weeks). Thirteen patients continued to remain on the study. The most common adverse events (occurring in 20% of patients) were fatigue, pyrexia, nausea, and diarrhea. To assess if more frequent dosing might maximize anti-tumor activity with acceptable tolerability, Bartlett et al. conducted the second multicenter, phase I, weekly dosing, dose-escalation study in 37 patients with refractory or recurrent HL or systemic ALCL [101]. The objective RR was 46% (16 of 35) with 29% of patients (10 of 35) achieving CR. Dose-limiting toxicities included grade 3 diarrhea and/or vomiting and grade 4 hyperglycemia. These remarkable results led to 2 phase II studies, one with Hodgkin disease and the other with systemic ALCL. In the first study of 102 patients with relapsed Hodgkin lymphoma, an overall RR of 75% was observed (76 of 102) with 34% (n = 35) CR’s [102]. In the second phase II study of 58 patients with relapsed systemic ALCL, overall RR of 86% (50 of 58) with 53% CR’s were noted [103]. These impressive results led to accelerated approval of brentuximab vedotin for the treatment of relapsed and refractory Hodgkin lymphoma and ALCL. This promising activity has led to several trials exploring the use of this agent in other CD30 expressing lymphomas including CTCL. MF and SS have variable expression of CD30 and hence a phase II single arm open label exploratory study evaluating the biologic effects of brentuximab vedotin in patients with CTCL is currently ongoing where patients regardless of their levels of CD30 expression (low, intermediate and high based on immunohistochemical studies) will be enrolled. In the future, there will be studies combining this with other modalities.

##### Other novel targetsPegylated liposomal doxorubicin (Caelyx^TM^)

Some patients with advanced-stage CTCL are treated with chemotherapy regimens like CHOP where response rates range between 40-70%. Pegylated Liposomal Doxorubicin (PLD) is an antineoplastic antibiotic with biologic activity similar to daunorubicin but with the added advantage of longer circulation time and higher intratumoral drug concentration levels leading to potentially higher target specificity and lower rate of infectious complications secondary to myelosuppression and cardiotoxicity. Dummer et al. conducted the EORTC 21012, phase II multicenter trial of PLD in 49 steroid naïve relapsed and refractory advanced MF patients that demonstrated a RR of 40.8% (20 of 29) including 6% CR’s (n = 3) and 34.7% PR (n = 17) [104]. The drug was considered safe for administration with the following ≥ grade 3 toxicity: 2% cardiac toxicity, allergy (2%), 4% constitutional symptoms, etc. No severe hematological toxicity or infections were seen. Duration of response and efficacy in SS patients are unknown. These results are encouraging and warrant further investigation.

##### Enzastaurin (LY317615)

Enzastaurin is a novel oral Protein kinase C (PKC) inhibitor that plays a role in cell survival, growth factor response, proliferation and angiogenesis. In vitro studies demonstrated apoptotic activity in two CTCL cell lines (HH and Hut-78) mediated via the AKT signaling and caspase pathways which led to its phase I trial in patients with advanced malignancies that established the MTD at 525 mg orally [105,106]. Querfeld at al. conducted the phase II multicenter trial of enzastaurin in patients with relapsed/refractory MF (IB-IVB) and SS [107]. Twenty-five patients were enrolled and 1 PR was observed in 1 MF patient with an overall RR of 5% by composite response evaluation. There were no CR’s. Although 17 patients with MF and 4 patients with SS had SD, the median time to progression (TTP) was only 78 days in MF patients and 44 days in SS patients. Accrual was terminated early due to lack of response. About 25% of the enrolled patients had meaningful pruritus relief. The authors concluded that it might be worth combining enzastaurin with other active agents to enhance its efficacy given its favorable toxicity profile.

##### TLR9 agonist vaccine

Toll-like receptor (TLR) agonists represent a novel mechanism of stimulating and enhancing the host’s innate and adaptive immunity to the skin homing tumor cells. Phase I/II studies in indolent B-cell lymphoma demonstrated that when local radiation to expose tumor antigens was combined with intratumoral injection of TLR-9 activating CpG-oligodeoxynucleotide (to serve as an in situ vaccination maneuver), meaningful clinical responses were observed in 4 of 15 patients (27%) with one attaining CR and safe tolerability [108]. The same approach was then applied to patients with MF in a phase I/II trial by Kim et al. where 15 patients with relapsed/refractory disease were treated with local low dose radiation followed by immediate and weekly intratumoral injections of CpG enriched oligodeoxynucleotide with assessment of response by mSWAT score [109]. The treatment was very well tolerated with only 1 grade 3 AE that included focal skin necrosis due to injection site reaction. Five of the 14 evaluable patients demonstrated a PR with 1 near PR mounting an overall RR close to 35% thereby demonstrating that in situ vaccination strategy combined with low dose radiation in MF warrants further investigation as it is a feasible option with acceptable toxicity.

##### Immunomodulatory agents

Lenalidomide (3-(4′aminoisoindolin-1′-one)-1-piperidine-2, 6-dione (Revlimid®) is one of the novel immunomodulatory (IMiDs) agents which was granted FDA approval for treatment of myelodysplastic syndromes associated with 5q deletion cytogenetic abnormality and relapsed/refractory multiple myeloma. IMiDs are structural and functional analogs of thalidomide that were specifically designed to enhance immunomodulatory and anticancer properties with better tolerability profiles. Lenalidomide, a second generation IMiD, was created using thalidomide as a template by adding an amino group to the 4th carbon of the phthaloyl ring and removal of a carbonyl group. This novel drug has several proposed mechanisms of action including immunomodulatory, antiangiogenic, and direct apoptotic properties, which culminate in cancer cell death either through direct interference with key functions of tumor cells or indirectly through modulation of signaling pathways that regulate their interaction to bone marrow stromal cells. While recent preclinical and clinical studies put forward a dual mechanism of action for lenalidomide, involving both a direct tumoricidal activity and immunomodulation. It is difficult to assign a specific mode of action to any specific tumor type. PK data indicates that the mean half-life of elimination increases with increasing dose, from approximately 3 h at the 5 mg dose up to approximately nine hours at the 400 mg dose. Steady state levels are achieved by day 4. Cytopenias are the primary adverse events associated with the administration of lenalidomide, particularly in subjects with compromised bone-marrow. However, these are manageable with dose interruptions and reductions. This agent has been studied in lymphoproliferative disorders. Querfeld et al. conducted a phase II trial of lenalidomide in relapsed/refractory MF and SS patients where 15 patients were treated with 25 mg of oral lenalidomide daily for 21 days followed by 1 week of rest [110]. Response was assessed after every cycle using Composite Assessment (CA) of Index Lesion Disease Severity for skin lesions, absolute Sézary cell count for quantification of circulating malignant lymphocytes and/or CT scans for evidence of adenopathy or visceral disease. Five patients achieved a partial response (defined as a CA ratio less than or equal to 0.5 with no new clinically abnormal lymph nodes, no progression of existing clinically abnormal lymph nodes, and no new cutaneous tumors). Six patients demonstrated minor responses such as regression of cutaneous tumor lesions, improved lymphadenopathy, and skin improvement from initial generalized erythroderma to less severe erythema with less scaling. Four patients experienced progression of disease. The most common side effects were anemia, fatigue/malaise, skin burning, pruritus, and lower leg edema. An initial flare phenomenon (manifested by increase in skin lesions, lymph node swelling, increase in blood tumor burden etc.) the biological basis of which is poorly understood was noted in almost all patients during the first cycle or beginning of each cycle with subsequent resolution during the course of treatment. This study while limited by the number of patients revealed encouraging activity and tolerable toxicity profile. Its role as monotherapy in relapsed/refractory CTCL is further being assessed in a larger single institution phase II study using updated and standardized clinical endpoint criteria such as the mSWAT. Given the chronicity of CTCL treatment and its established potential in multiple myeloma, its role as a maintenance regimen following systemic chemotherapy (gemcitabine or doxil) in advanced stage CTCL patients is being explored in a pivotal EORTC initiated phase III trial.

## Conclusions

At present there are many promising agents with activity against cutaneous T-cell lymphomas as summarized in Table 1. However establishing a definitive diagnosis, accurate staging, risk stratification and selection of appropriate initial therapy still remains critical. High response rates and favorable toxicity profile of HDAC inhibitors has become the cornerstone of treatment of advanced and relapsed/refractory CTCL. Future clinical trials would focus on utilizing these novel effective agents early in the course of this neoplasm thereby forming a backbone to which sequential therapies could be added in an escalated rational step-wise approach. Designing and implementing well designed clinical trials with careful thought to correlative molecular studies will become the dogma of clinical investigators and will equip clinicians to deliver personalized treatment. This hopefully will help move the field forward towards a cure of these diseases, as treatment currently remains largely palliative for most patients with CTCL.

**Table 1:** Summary of Major Clinical Trials of Novel Drugs in CTCL

Agent	Target	Type of Study	Dose	No of Patients	Stage of Patients	ORR [CR +PR] (%)	CR (%)	PR (%)	Median DoR^1^	Status in CTCL	Major side- effects	Reference
Vorinostat	Hydrox-amic acid Class I, II, IV HDAC inhibitor	Phase II	400mg oral/d	74	IB=11	29.7	1.35	28.35	Not reached	Approved for R/R^2^ CTCL	Diarrhea fatigue, nausea, anorexia, changes in taste, thrombocyto-penia, eight decrease	[35]
					IIA=2							
					IIB=19							
					III=20							
					IVA=18							
					IVB=4							
Romi- depsin	Cyclic peptide Class I, II, IV HDAC inhibitor	Phase II	14 mg/m2 on days 1, 8, 15 every 28days	71	IA=1	33	7	26	13.7 months	Approved for R/R^2^ CTCL	Fatigue, nausea, thrombocyto-penia, anemia, Hypocal-cemia	[40]
		NCI-1312										
					IB=6							
			IV		IIA=2							
					IIB=15							
					IIIA=3							
					IIIB=3							
					IVA=28							
					IVB=13							
		Phase II	14 mg/m2 on days 1, 8, 15 every 28days	96	IB=15	34	6	28	15 months	Approved for R/R^2^ CTCL	Nausea, asthenia, vomiting, anorexia	[41]
					IIA=13							
		GPI-04-0001										
					IIB=21							
			IV		III=23							
					IVA=24							
Belinostat	Hydrox-amic acid Class I, II, IV HDAC inhibitor	Phase II	1000 mg/m2 on days, 1-5 every 3weeks	29	IV=15	14	7	7	9.1 Months	Clinical activity but not approved	Neutro-penia, thrombocyto-penia pruritis, rash, edema, adynamic ileus	[53]
			IV									
Panobio- stat	Hydrox-amic acid Class I, II, IV HDAC inhibitor	Phase II	20mg orally on days 1,3,5 every 28 days	95	≥ IIB=68	15.78	2	13.78	Not reached	Clinical activity but not approved	Diarrhea, thrombocyto-penia, nausea, pruritus, fatigue, asthenia	[55]
Prala- trexate	RFC-1	Phase I	Dose finding study	29 at optimal dose of 15 mg/m2 weekly x 3 every 4 weeks IV	Data not available	43	Un-known	Un-known	Not reached	Clinical activity and acceptable toxicity at MTD.	Fatigue, nausea, mucositis, edema, epistaxis	[65]
Bortez- omib	Various	Phase II	1.3 mg/m2 on days 1,4, 8 and 11 every 21 days	10	IIIA=1	70	10	60	9 months	Clinical activity but not approved	Neutropenia, thrombocyto-penia, sensory neuropathy	[76]
					IIIB=3							
					IVA/B=6							
			IV									
Foro- desine	Inhibits purine nucleoside phophoryla-se	Phase I	Dose finding study 40–320 mg/m^2^ oral daily	9	≥ III=8	55	22	33	Not reached	On-going Phase II trial in CTCL	Nausea, fatigue, dyspnea, edema, urinary casts	[81]
Alemtu- zumab	Mono-clonal anti-body against CD52	Phase II	Escalating dose up to 30 mg thrice/week	22	IIA=1	52	21.4	14.2	Not reached	Approved for use in CTCL	Fever, rigors, nausea, fatigue, hypotension	[85]
					IIB=2							
					IIIA=6							
					IIIB=4							
					IVA=7							
			IV									
					IVB=2							
SGN-35	Anti-CD30 combined with cytotoxic agent auri-statin	Phase II in R/R^2^ Hodgkin lymphoma after ASCT^3^	1.8 mg/kg every 3 weeks IV	102	Not applicable	75	34	Unknown	Not reached	On-going Phase II trial in CTCL	Neuropathy, nausea, fatigue, neutropenia, diarrhea	[100]
		Phase II in R/R sALCL^4^	1.8 mg/kg every 3 weeks	58	Not applicable	86	53	Unknown	Not reached	On-going Phase II trial in CTCL	Neuropathy, nausea, fatigue, neutropenia, diarrhea	[101]
			IV									
TLR9 agonist vaccine	Enhance host immune response	Phase II	Intra-tumoral injection with radiation	14	IB=5	35	None	28.5	Not reached	Clinical activity but not approved	Well tolerated	[109]
					IIB=10							
Lena- lidomide	IMiD	Phase II	25 mg orally daily for 21 days followed by 1 week of rest	15	Not available	30	None	30	Not reached	On-going larger Phase II trial in CTCL	anemia, fatigue malaise, skin burning, pruritus, lower leg edema, initial flare of disease	[110]

## Competing interests

Author 1 Salvia Jain: Has no financial disclosures or competing interests.

Author 2 Jasmine Zain: I consult for and have done speaking programs on behalf of Allos Theraperutics and Seattle Genetics. However, none of these companies have provided any financing of this manuscript and will not gain financially form the publication of this manuscript. I hold no stocks or shares or patents in any organization related to this manuscript and have no financial or non- financial competing interests.

Author 3 Owen O’Connor: *Celgene:* Consultancy, Research Funding; *Merck:* Research Funding; *Novartis:* Research Funding; *Spectrum:* Research Funding.

## Authors’ contributions

SJ, JZ and OOC drafted the manuscript. All authors read and approved the final manuscript.
